# Effect of genetic background on the cardiac phenotype in a mouse model of Emery-Dreifuss muscular dystrophy

**DOI:** 10.1016/j.bbrep.2019.100664

**Published:** 2019-07-12

**Authors:** Nicolas Vignier, Nathalie Mougenot, Gisèle Bonne, Antoine Muchir

**Affiliations:** a Sorbonne Université, INSERM UMRS974 Centre de Recherche en Myologie, Institut de Myologie, G.H. Pitié Salpêtrière, F-75651, Paris Cedex 13, France; b Sorbonne Université, INSERM UMS28 Phénotypage du petit animal, Faculté de Médecine Pierre et Marie Curie, F-75013, Paris, France

**Keywords:** *LMNA*, A-type lamins, Cardiomyopathy, Mouse models, Genetic background

## Abstract

A-type lamins gene (*LMNA*) mutations cause an autosomal dominant inherited form of Emery-Dreifuss muscular dystrophy (EDMD). EDMD is characterized by slowly progressive muscle weakness and wasting and dilated cardiomyopathy, often leading to heart failure-related disability. EDMD is highly penetrant with poor prognosis and there is currently no specific therapy available. Clinical variability ranges from early onset with severe presentation in childhood to late onset with slow progression in adulthood. Genetic background is a well-known factor that significantly affects phenotype in several mouse models of human diseases. This phenotypic variability is attributed, at least in part, to genetic modifiers that regulate the disease process. To characterize the phenotype of A-type lamins mutation on different genetic background, we created and phenotyped C57BL/6JRj-*Lmna*^H222P/H222P^ mice (C57^*Lmna*^^p.H222P^) and compared them with the 129S2/SvPasCrl-*Lmna*^H222P/H222P^ mice (129^*Lmna*^^p.H222P^). These mouse strains were compared with their respective control strains at multiple time points between 3 and 10 months of age. Both contractile and electrical cardiac muscle functions, as well as survival were characterized. We found that 129^*Lmna*^^p.H222P^ mice showed significantly reduced body weight and reduced cardiac function earlier than in the C57^*Lmna*^^p.H222P^ mice. We also revealed that only 129^*Lmna*^^p.H222P^ mice developed heart arrhythmias. The 129^*Lmna*^^p.H222P^ model with an earlier onset and more pronounced cardiac phenotype may be more useful for evaluating therapies that target cardiac muscle function, and heart arrhythmias.

## Highlights

• Mouse model of Emery-Dreifuss muscular dystrophy generated on 129S2/svPasCrl genetic background have a greater life expectancy.
• Mouse model of Emery-Dreifuss muscular dystrophy generated on 129S2/svPasCrl genetic background showed exacerbated arrhythmia susceptibility.
• Mouse model of Emery-Dreifuss muscular dystrophy generated on 129S2/svPasCrl genetic background showed more pronounced dilated cardiomyopathy.

## 1 Introduction

Emery-Dreifuss muscular dystrophy (EDMD) is characterized by the clinical triad of i/slowly progressive muscle weakness and wasting in a scapulo-humeroperoneal distribution; ii/early contractures of the elbows, ankles, and posterior neck; and iii/cardiac conduction defects associated with dilated cardiomyopathy [1]. *LMNA* mutations encoding nuclear A-type lamins are responsible for the autosomal forms of EDMD [2]. Genetically engineered mouse models of EDMD have brought valuable insights in our understanding of the molecular mechanisms of the disease [3]. They have been instrumental in the identification of signaling pathways responsible for the cardiac dysfunction and have provided invaluable tools for proposing novel treatment for this disease [[4], [5], [6], [7], [8], [9], [10]]. The knock-in *Lmna*^p.H222P/H222P^ mice mouse model carrying the *LMNA* p.H222P mutation previously described in patient with classical form of EDMD [11], recapitulates all the features of EDMD [12]. In particular, *Lmna*^H222P/H222P^ mice developed a dilated cardiomyopathy associated with cardiac conduction defects [11,13].

*Lmna*^H222P/H222P^ mice were generated using a C57BL/6 background, an inbred strain and useful reductionist tool to study effects of single gene mutations, However in the clinic, *LMNA* mutations show a strong degree of pleiotropy and therefore understanding how genetic background influences this is likely to prove important. It is known that phenotypic variation that arises due to the influence of genetic background can be of importance to genetically engineered mouse models [14,15]. More recently, it has been showed that the genetic background was a determining factor for the severity of muscular dystrophy in a mouse model of Duchenne muscular dystrophy [16,17]. To investigate the effect of genetic background on the cardiac involvement of EDMD, we have backcrossed *Lmna*^p.H222P/H222P^ mice to different pure genetic background, C57BL/6JRj (C57^*Lmna*^ ^p.H222P^) and 129S2/SvPasCrl (129^*Lmna*^ ^p.H222P^). We found that cardiac involvement was more pronounced in the 129^*Lmna*^ ^p.H222P^ mouse strain compared with the C57^*Lmna*^ ^p.H222P^ mouse strain, suggesting that genetic background significantly modifies the severity of dilated cardiomyopathy linked to *LMNA* mutations.

## 2 Results

### 2.1 Genetic characterization of mouse strains

**Fig. 1:**
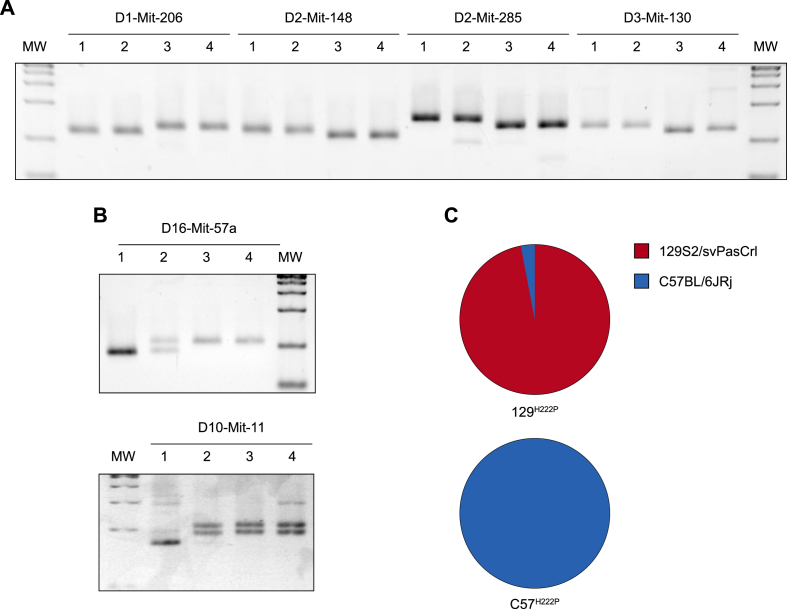
Genomic characterization of the genetic background of 129^*Lmna*^^p.H222P^ and C57^*Lmna*^^p.H222P^ mice (**A**) Representative discrimination of Simple Sequence Length Polymorphisms (SSLP) between 129S2/svPasCrl [1], 129^*Lmna*^^p.H222P^ [2], C57BL/6JRj [3] and C57^*Lmna*^^p.H222P^ [4] mouse strains. (**B**) Discrimination of a heterozygous and homozygous SSLP in 129^*Lmna*^^p.H222P^ [2] mouse strain in comparison with 129S2/svPasCrl [1], C57BL/6JRj [3] and C57^*Lmna*^^p.H222P^ [4] mice strains. (**C**) Schematic representation the 129S2/svPasCrl (red) and C57BL/6JRj (blue) mouse strain background in 129^*Lmna*^^p.H222P^ and C57^*Lmna*^^p.H222P^ mouse strain.

To assess the genetic background of the C57^*Lmna*^ ^p.H222P^ and 129^*Lmna*^ ^p.H222P^ mouse strains, we used a marker-assisted selection protocol (MASP) based on genome-wide analysis of genetic polymorphisms, which allows the discrimination between different strains of mice [18]. We used simple sequence length polymorphism (SSLP) that correspond to short cytosine, adenine nucleotide tandem repeats (CA)*n*. The number *n* of (CA) repeats is specific for C57BL/6JRj or 129S2/SvPasCrl strains [19]. We screened for 50 SSLPs, covering the entire mouse genome, on all four mouse strains (Table S1). We observed that all the 50 SSLPs were homozygous and matched in length for both C57BL/6JRj and C57^*Lmna*^ ^p.H222P^ mice (Fig. 1A). Besides, we found that 48 of the 50 SSLPs were homozygous and matched in size in 129^*Lmna*^ ^p.H222P^ mice compared with 129S2/svPasCrl mice (Fig. 1A). Furthermore, one SSLP (D16-Mit-57a), located on chromosome 16, was heterozygous with one allele from C57BL/6JRj strain and the second from 129S2/SvPasCrl strain (Fig. 1B). Moreover, one SSLP (D10-Mit-11) in 129^*Lmna*^ ^p.H222P^ mice, located on chromosome 10, was homozygous for C57BL/6JRj strain. Hence, the C57^*Lmna*^ ^p.H222P^ mouse strain was entirely derived on C57BL/6JRj genetic background (Fig. 1C). Moreover, 97% of the 129^*Lmna*^ ^p.H222P^ strain was similar to 129S2/svPasCrl strain and the other 3% was similar to C57BL/6JRj strain (Fig. 1C).

### 2.2 Body weight

**Fig. 2:**
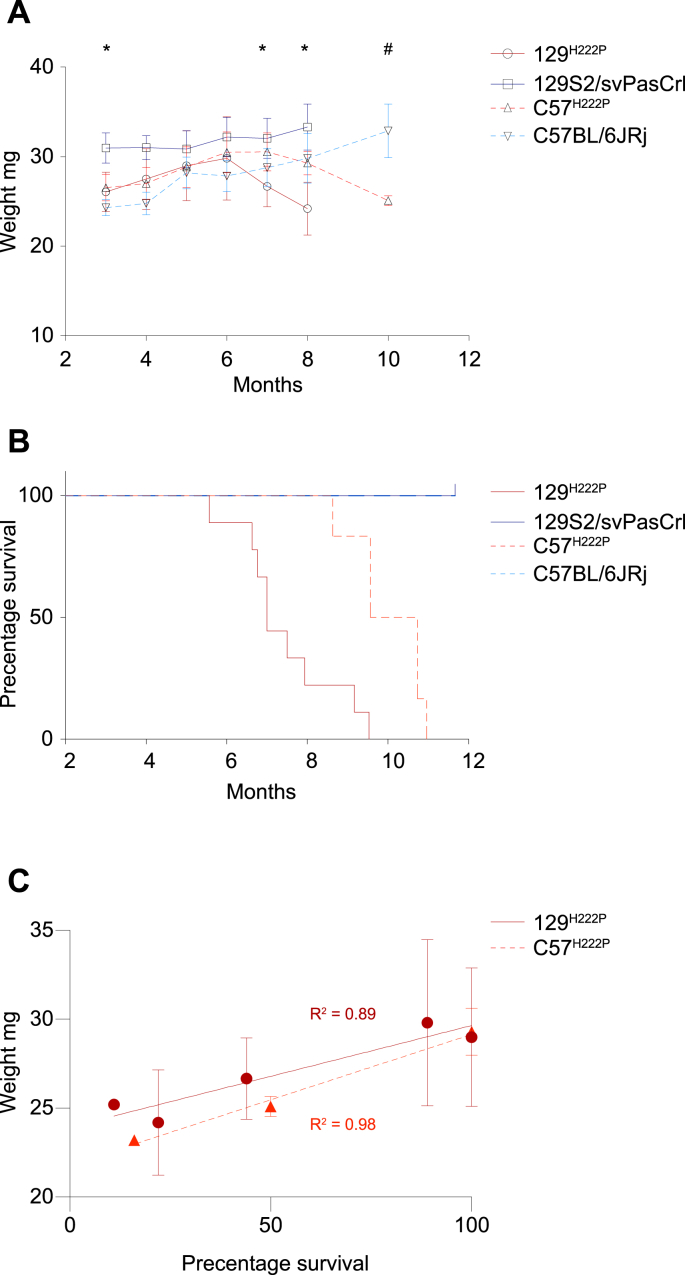
Effect of genetic background on the weight and the lifespan of 129^*Lmna*^^p.H222P^ and C57^*Lmna*^^p.H222P^ mice. (**A**) Weight curves for 129S2/svPasCrl (n = 12), 129^*Lmna*^^p.H222P^ (n = 9), C57BL/6JRj (n = 8), and C57^*Lmna*^^p.H222P^ (n = 6) mice. Values are presented as mean ± SD. Multiple group comparison was performed with Kruskal Wallis test with Dunn’s test post-test. *p ≤ 0.01 between 129^*Lmna*^ ^p.H222P^ mice and 129S2/svPasCrl mice. ^#^*p* ≤ 0.01 between C57^*Lmna*^ ^p.H222P^and C57BL/6JRj mice. (**B**) Kaplan–Meier survival curves for 129S2/svPasCrl (n = 12), 129^*Lmna*^^p.H222P^ (n = 9), C57BL/6JRj (n = 8), and C57^*Lmna*^^p.H222P^ (n = 6) mice. Survival curves comparison was performed with the logrank test (mantel-cox test). ^**^p ≤ 0.01 between C57^*Lmna*^^p.H222P^ and 129^*Lmna*^^p.H222P^ mice. ^####^*p* ≤ 0.0001 between 129^*Lmna*^^p.H222P^ mice and 129S2/svPasCrl mice. (**C**) Regression analysis of weight and survival percentage for 129^*Lmna*^^p.H222P^ and C57^*Lmna*^^p.H222P^ mice. Values are presented as mean ± SD.

The loss of body weight characterizes the progression of the disease in *Lmna*^H222P/H222P^ mice [12]. To follow the time course of the disease of the 129^*Lmna*^ ^p.H222P^ and C57^*Lmna*^ ^p.H222P^ mice, we weighed mice every month from 3 to 10 months of age. The 129^*Lmna*^ ^p.H222P^ mice started to lose weight from 6 months of age compared with 129S2/svPasCrl mice (Fig. 2A). The C57^*Lmna*^ ^p.H222P^ mice started to lose weight at 7 months of age compared with C57BL/6JRj mice (Fig. 2A). The median survival of the 129^*Lmna*^ ^p.H222P^ mice was significantly shorter compared to the C57^*Lmna*^ ^p.H222P^ mice, *p* < 0.001 (Fig. 2B). The median survival of the 129^*Lmna*^ ^p.H222P^ mice was 7 months of age, while the median survival of C57^*Lmna*^ ^p.H222P^ mice was 10 months. (Fig. 2B). The weight loss was correlated with decreased lifespan for both 129^*Lmna*^ ^p.H222P^ and C57^*Lmna*^ ^p.H222P^ mice. The coefficient determination R^2^ between weight and percentage of survival was 0.89 for 129^*Lmna*^ ^p.H222P^ mice and 0.98 for C57^*Lmna*^ ^p.H222P^ mice (Fig. 2C). These results suggested that the phenotype of 129^*Lmna*^ ^p.H222P^ mice was more severe than for the C57^*Lmna*^ ^p.H222P^ mice.

### 2.3 Cardiac structure and function

**Fig. 3:**
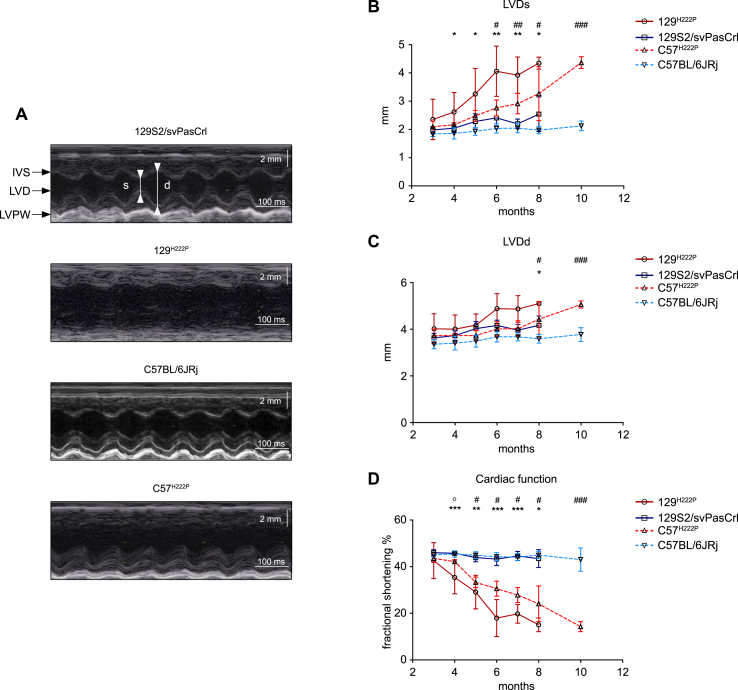
Effect of genetic background on the 129^*Lmna*^^p.H222P^ and C57^*Lmna*^^p.H222P^ mice cardiac function. (**A**) Representative echocardiographic M-mode images at 5 months of age from 129S2/svPasCrl, 129^*Lmna*^^p.H222P^, C57BL/6JRj and C57^*Lmna*^^p.H222P^ mice. (**B**) Graph representing the left-ventricular end systolic diameters (LVDs) from 129S2/svPasCrl, 129^*Lmna*^^p.H222P^, C57BL/6JRj and C57^*Lmna*^^p.H222P^ mice from 2 to 12 months of age. Values are presented as mean ± SD. Multiple group comparison was performed with Kruskal Wallis test with Dunn’s test post-test. (**C**) Graph representing the left-ventricular end systolic diastolic (LVDd) from 129S2/svPasCrl, 129^*Lmna*^^p.H222P^, C57BL/6JRj and C57^*Lmna*^^p.H222P^ mice from 2 to 12 months of age. Values are presented as mean ± SD. Multiple group comparison was performed with Kruskal Wallis test with Dunn’s test post-test. (**D**) Graph representing the fractional shortening (FS) from 129S2/svPasCrl, 129^*Lmna*^^p.H222P^, C57BL/6JRj and C57^*Lmna*^^p.H222P^ mice from 2 to 12 months of age. Values are presented as mean ± SD. Multiple group comparison was performed with Kruskal Wallis test with Dunn’s test post-test.*p ≤ 0.01, **p ≤ 0.001 and ***p ≤ 0.0001 between 129^*Lmna*^^p.H222P^ mice and 129S2/svPasCrl mice.^#^*p* ≤ 0.01, ^##^*p* ≤ 0.001 and ^###^*p* ≤ 0.0001 between C57^*Lmna*^^p.H222P^ and C57BL/6JRj mice. ^o^*p* ≤ 0.01 between C57^*Lmna*^^p.H222P^ and 129^*Lmna*^^p.H222P^ mice.

Cardiac structure and function were determined by echocardiography in the four different groups of mice. Compared with 129S2/svPasCrl mice, 129^*Lmna*^ ^p.H222P^ mice had significantly increased left-ventricular end systolic diameter, starting at 4 months of age (Fig. 3A and C, Table S2). The left-ventricular end diastolic diameters started to be significantly increased in 129^*Lmna*^ ^p.H222P^ mice at 8 months of age (Fig. 3A and B, Table S2). Compared with C57BL/6JRj mice, C57^*Lmna*^ ^p.H222P^ mice had significantly increased left-ventricular end systolic and left-ventricular end diastolic diameters at 6 months of age (Fig. 3A and C, Table S2). We observed a statistically significant decreased fractional shortening (FS) for 129^*Lmna*^ ^p.H222P^ mice compared with wild type 129S2/svPasCrl animals at 4 months of age (Fig. 3D, Table S2), while it was noticeable at 5 months of age for C57^*Lmna*^ ^p.H222P^ mice. Taken together, these results showed a more severe cardiac phenotype in the 129^*Lmna*^ ^p.H222P^ mice compared with the C57^*Lmna*^ ^p.H222P^ animals.

### 2.4 Conduction defects and arrhythmias

**Fig. 4:**
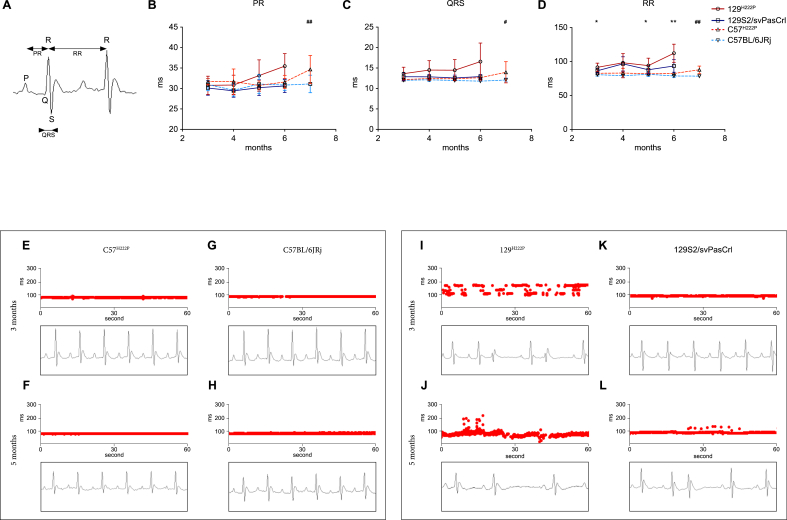
Effect of genetic background on the cardiac conduction and arrhythmias (**A**) Representation of electrocardiogram (ECG) trace with the P wave, the QRS complex, the PR interval and the RR interval. (**B**) Graphs showing PR interval, for the 129^*Lmna*^^p.H222P^ mice, 129S2/svPasCrl mice, C57^*Lmna*^^p.H222P^ and C57BL/6JRj mice. Values are presented as mean ± SD. Multiple group comparison was performed with Kruskal Wallis test with Dunn’s test post-test. (**C**) Graphs showing QRS interval, for the 129^*Lmna*^^p.H222P^ mice, 129S2/svPasCrl mice, C57^*Lmna*^^p.H222P^ and C57BL/6JRj mice. Values are presented as mean ± SD. Multiple group comparison was performed with Kruskal Wallis test with Dunn’s test post-test. (**D**) Graphs showing RR interval, for the 129^*Lmna*^^p.H222P^ mice, 129S2/svPasCrl mice, C57^*Lmna*^^p.H222P^ and C57BL/6JRj mice. Values are presented as mean ± SD. Multiple group comparison was performed with Kruskal Wallis test with Dunn’s test post-test. (**E-L**) Variation of the RR interval from the four mouse strains at 3 and 5 months. *p ≤ 0.05 and **p ≤ 0.005 between 129^*Lmna*^^p.H222P^ mice and 129S2/svPasCrl mice. ^#^*p* ≤ 0.05 and ^##^*p* ≤ 0.005 between C57^*Lmna*^^p.H222P^ and C57BL/6JRj mice.

Given that conduction defects and arrhythmias have been previously described in *Lmna*^H222P/H222P^ mice [12,13], we set out to assess of the cardiac electrical conduction disturbances (Fig. 4A) in the four different groups of mice. The PR interval and the QRS complex duration were extended, without reaching significance (Table S4), in 129^*Lmna*^ ^p.H222P^ mice and C57^*Lmna*^ ^p.H222P^ mice compared with 129S2/svPasCrl mice and C57BL/6JRj mice, respectively (Fig. 4B and C). Both PR interval and QRS complex increases were more pronounced in the 129^*Lmna*^ ^p.H222P^ mice than in the C57^*Lmna*^ ^p.H222P^ mice, with an earlier onset in 129^*Lmna*^ ^p.H222P^ mice (Table S3). The RR interval was significantly prolonged in the 129^*Lmna*^ ^p.H222P^ mice at 3, 5 and 6 months of age compared with 129S2/svPasCrl mice (Fig. 4D, Table S3). Similarly, RR interval was also lengthened in the C57^*Lmna*^ ^p.H222P^ mice at 6 months compared with C57BL/6JRj mice (Fig. 4D, Table S3). Furthermore, ECG analyses revealed that neither C57BL/6JRj mice nor C57^*Lmna*^ ^p.H222P^ mice develop heart arrhythmias at 3 or 5 months of age (Fig. 4E, F, 4G, 4H). However, 129^*Lmna*^ ^p.H222P^ mice developed heart arrhythmia (supraventricular premature contractions and sino ventricular blocks) at 3 (Figure 4I) and 5 months of age (Fig. 4J). We noted that 129S2/svPasCrl mice did not have heart arrhythmia at 3 months of age (Fig. 4K) but developed some by 5 months of age (Figure 4L). Taken together these results showed a higher susceptibility to conduction defects in 129^*Lmna*^ ^p.H222P^ mice.

## 3 Discussion

The phenotype of a given single-gene mutation in genetically engineered mouse models is modulated by the genetic background. This effect is attributable to modifier genes, which function in combination with the causative gene. Developing congenic mouse strains not only helps us to investigate the effect of *LMNA* mutations on phenotype but would also facilitate the development of mouse models that more accurately mimic certain features of EDMD. Using validation of single sequence length polymorphism, a method that can be routinely performed, we demonstrated that this approach could be successfully used to certify inbred strains of mouse models of muscular dystrophies. We showed here significant strain-dependent differences in cardiac function between inbred strains of *Lmna*^p.H222P/H222P^ mice. Particularly, the cardiac phenotype was more pronounced in the 129S2/svPasCrl genetic background. These findings demonstrate the complex effects of genetic divergence between inbred strains on cardiac functions in mouse models of EDMD.

Unlike the C57^*Lmna*^ ^p.H222P^ mice, we found that 129^*Lmna*^ ^p.H222P^ mice showed exacerbated arrhythmia susceptibility. This is in agreement with previously reported findings that C57BL/6J mice are resistant to arrhythmia [20,21]. It is known that genetic background dominates the susceptibility to ventricular arrhythmias [22]. In this context, this should be considered when studying cardiac electrical activity in mice. Further exploration of the genetic differences between the two congenic mouse strains, which account for the difference in the cardiac phenotypes of the same *Lmna* p.H222P mutation, is warranted. Indeed, genomic analysis of the 129^*Lmna*^ ^p.H222P^ mice and C57^*Lmna*^ ^p.H222P^ mice will be of interest to identify specific locus associated to arrhythmia. Such a study may provide new mechanistic and clinically relevant insight into the alteration of cardiac function between these strains. Moreover, the relatively sensitive 129S2/svPasCrl mice would be of great value to investigate the genetic basis underlying ventricular arrhythmias. In conclusion the 129^*Lmna*^ ^p.H222P^ mouse cardiac phenotype was more deleterious than in C57^*Lmna*^ ^p.H222P^ mouse, at least in part because genetic background of 129S2/svPasCrl present a predisposition for arrhythmia. In human EDMD, arrhythmia-related symptoms frequently preceded heart dilation and heart failure symptoms ([23]).

In summary, our data suggest that 129^*Lmna*^ ^p.H222P^ mice have a severe progressive cardiac muscle disease with early-onset deficits. Thus, this mouse model may be a particularly suitable model for evaluating therapeutic strategy for EDMD.

## 4 Material and methods

### 4.1 Mouse strains

*Lmna*^H222P/H222P^ mice [12] were backcrossed eight times with the congenic strains C57BL/6JRj and 129S2/svPasCrl (Janvier Labs). The animals were fed chow and housed in a disease-free barrier facility at 12 h/12 h light/dark cycles. The French Ministry of Health has approved all animal experiments (approval number #00982.03). Accredited personnel dedicated to the Care and Use of Experimental Animals has conducted all animal experiments (accreditation number #75–679). The animal experiments were performed according to the guidelines from Directive 2010/63/EU of the European Parliament on the protection of animals used for scientific purposes.

### 4.2 Simple Sequence Length Polymorphisms screening

Genomic DNA was extracted from mouse tail samples digested in 0.4 mg/ml proteinase K digestion buffer (Tris pH 8,5 100 mM, EDTA 5 mM, SDS 0,2%, NaCl 200 nM), precipitated by 100% isopropanol and suspended in water at 500 ng/μl final concentration. Polymerase chain reaction (PCR) was performed using AmpliTaq Gold™ 360 Master Mix according to the provider recommendations. PCR cycle were 95 °C for 10 mn, 10 cycles (95 °C for 10 s, 60 °C for 10 s, 72 °C for 10 s), 72 °C for 5 mn. PCR products were analyzed in 5% Nuseive CTG agarose gel.

### 4.3 Echocardiography

Mice were anesthetized with 0.75% isoflurane in O_2_ and placed on a heating pad (25 °C). Echocardiography was performed using an Vivid7 ultrasound with an 11 MHz transducer applied to the chest wall. Cardiac ventricular dimensions and fractional shortening were measured in 2D mode and M-mode, 3 times per animal. A blinded experimenter, unaware of the genotype performed the examinations.

### 4.4 Electrocardiography

Electrocardiograms were recorded from living mice using the non-invasive ecgTUNNEL^®^ (Emka Technologies). Waveforms were recorded using Iox Software v1.8.9.18 and intervals were measured with ecgAUTO v1.5.12.50, using the average of three representative consecutive beats. A blinded experimenter, unaware of the genotype performed the examinations.

### 4.5 Statistics

Statistical analyses were performed using GraphPad Prism software. Statistical significance between groups of mice was analyzed with a corrected parametric test, Welch’s *t*-test when compared two sets of data, or Kruskal Wallis test with Dunn’s test post-test when compared multiple sets data, with a value of *P* ≤ 0.05 being considered significant. To validate results of echocardiographic analyses, we performed a non-parametric test (Wilcoxon-Mann-Whitney test). Survival curves were generated using the method of Kaplan and Meier, and survival curves comparison was performed with the log rank test (mantel-cox test). Regression analysis was performed for a confidence interval of 95%.

## Competing interests

All other authors have declared no conflicts of interest.

## Conflict of interest

None for all the authors.

## Author contribution

Conceptualization, A.M. and N.V.; Investigation, N.V. and N.M.; Writing – Original Draft, A.M; Writing – Review & Editing, N.V., G.B., and A.M.; Funding Acquisition, A.M.; Supervision, A.M.
