# Supplementary material for: Effect of genetic background on the cardiac phenotype in a mouse model of Emery-Dreifuss muscular dystrophy
Source: Biochem Biophys Rep. 2019 Jul 12;19:100664. doi: 10.1016/j.bbrep.2019.100664 (PMC6630059; doi:10.1016/j.bbrep.2019.100664)
Supplement: Multimedia component 2 [file mmc2.docx]

**Supplemental data**

Four tables

**Table S1: Simple sequence length polymorphism primers**

| Markers ID | Chromosome N° | PCR size product | | size difference | Forward Primer | Reverse Primer |
| --- | --- | --- | --- | --- | --- | --- |
|  |  | 129S2/SvPasCrl | C57BL/6JRj |  |  |  |
| D1 Mit 206 | 1 | 119 | 127 | 8 | TGAGGCACCTTTGTATTCAGC | CCAGATGTCTTTGAACATTCTCC |
| D1 Mit 211 | 1 | 160 | 150 | -10 | GTTATTCATCAAAATACAGATGGCC | TCTGCTGCTAAGTAGAATGAATGC |
| D1 Mit 303 | 1 | 120 | 135 | 15 | GGTTTCTATTTCGGTTCTCGG | TCTGTGCTGCAAAACAGAGG |
| D2 Mit 148 | 2 | 130 | 120 | -10 | GTTCTCTGATCTACGGGCATG | TTCACTTCTACAAGTTCTACAAG |
| D2 Mit 206 | 2 | 120 | 160 | 40 | TGTCAGAACTGGACAATGTCG | ATGATAACAGACACTAATGATTAG |
| D2 Mit 285 | 2 | 155 | 140 | -15 | TCAATCCCTGTCTGTGGTAGG | TATGACACTTACAAGGTTTTTGGTG |
| D2 Mit 312 | 2 | 120 | 135 | 15 | TGAGAGTCTCTCCATTGGTATAAGG | TTCTCTGTGTCCCAACTTCTTG |
| D3 Mit 130 | 3 | 140 | 120 | -20 | AACACATGAAACGTGTGCG | TGATAGGCATGCTTAAGC |
| D3 Mit 258 | 3 | 190 | 210 | 20 | ACACAGGAATATGGTGTGAGTCC | AACAGTAGTAGAGGCAAGTAG |
| D3 Mit 278 | 3 | 100 | 120 | 20 | AACTACCATCTAAAACATCCTCTGTG | AGATCCCTAGAGAAACAGAACTGG |
| D3 Mit 44 | 3 | 140 | 160 | 20 | CCTGACTCATTTATTTAACTCC | CCCTATCACATAGGGCAACC |
| D4 Mit 166 | 4 | 180 | 210 | 30 | AGTTTCCTTTCTCTTCTACTTG | AGGGCATAGGAAACTTTCAGG |
| D5 Mit 239a | 5 | 160 | 145 | -15 | ATTGCAGACATAAAGGATATTTTGG | GCCAGCCTGGCTTACATAAG |
| D5 Mit 24 | 5 | 190 | 170 | -20 | CACTTGCCACACAGCAGG | CGTGCATGCACTAGTGTGTG |
| D5 Mit 388 | 5 | 180 | 190 | 10 | TTTCAGAGGGTGGGAGGTA | CCTGGACTCATGGAAGCATT |
| D6 Mit 102 | 6 | 210 | 160 | -50 | CCATGTGGATATCTTCCCTTG | GTATACCCAGTTGTAAATCTTGT |
| D6 Mit 159a | 6 | 140 | 120 | -20 | CATATTCAAGACGGAGACTAGTTCC | CACATGAAACACATGCACACA |
| D6 Mit 166 | 6 | 120 | 100 | -20 | CATTTTATTTTATTGATGGATGTGTG | GTTGTCTTATGGCTGCCATG |
| D6 Mit 201 | 6 | 120 | 150 | 30 | TGCTTCCTCTCTGCTGTAAGC | AACTAAGGCCAGTACTGAAAAGTACA |
| D7 Mit 145 | 7 | 210 | 150 | -60 | CAGGTGACCTTGGTCATGG | AGAGCCCAGGGGTTTTAAG |
| D7 Mit 246 | 7 | 160 | 150 | -10 | CACACAAAGCCGCAGTTCTA | TTGTTACGTGGCCTAGATTGG |
| D7 Mit 253 | 7 | 110 | 90 | -20 | TGTGGGTGCAACCAAATG | TTTGGTGATATAGATACTAGGTGTGTG |
| D8 Mit 121 | 8 | 230 | 240 | 10 | CGGTCAATCCCGAGTTTG | CAAGGCTGTCAGTCAGTGTAGG |
| D8 Mit 242 | 8 | 210 | 180 | -30 | TGTGCAACCAATTTCTTCCA | CCCATGATTTATTCAGACTGAGG |
| D9 Mit 269 | 9 | 160 | 190 | 30 | GGACTAATAGTCAACTGTGTAA | AGGAAGACTGAAAACTTGTG |
| D9 Mit 311a | 9 | 160 | 140 | -20 | CAGAAAACACTAATGTTTTGTATC | AGAAAACCACCTACCTACC |
| D10 Mit 11 | 10 | 160 | 190 | 30 | GAGAAGTCACTGGGAGCTGG | TTGCCAGGTTGCTCTTCTTT |
| D10 Mit 2 | 10 | 130 | 120 | -10 | CTGCTCACAACCCATTCCTT | GTTCATTTGAGGCACAAGCA |
| D10 Mit 38 | 10 | 190 | 160 | -30 | CGATGAGCCCTAACACCAAT | CCTGTTACAAACTAAACCAAAC |
| D11 Mit 219 | 11 | 150 | 130 | -20 | TTGTATGTATAGATGCATTTGAATGG | GGTTTGTATAAATTCTCACCTGTGC |
| D11 Mit 224a, | 11 | 155 | 145 | -10 | CATCATGGAAGGAGAGAAC | CTATAATAATTTCTCCAACCTC |
| D12 Mit 12 | 12 | 150 | 140 | -10 | TTCAATGCCTTCTGGCTTCT | GATTACCGGGTGTGTGACCT |
| D12 Nds2 | 12 | 180 | 200 | 20 | ACATGGTAATTTATGGGCAA | CTGGATACCTGCAATAGTAGA |
| D13 Mit 77 | 12 | 260 | 270 | 10 | TCTTTGAAGTCCCTTTCAAAGC | ATAGCACTGCACTCATGCTCA |
| D14 Mit 132 | 14 | 125 | 130 | 5 | GAACAGCACCATCCACACAC | GTGGGGTTATATGCAGATACTCG |
| D14 Mit 8 | 14 | 200 | 190 | -10 | TCACAGGTGCTCTCAGTCATG | GCAAATACTTCCCTTCTTGGG |
| D15 Mit 13a,b | 15 | 120 | 135 | 15 | GGAGACAAAAATGAACTCCTGG | TTGTAAGACAAGCATAGCTCAACA |
| D15 Mit 193 | 15 | 110 | 130 | 20 | TTGTGTAAGCCAATCTAATAAAGCC | GTTGCTGTGCTCATGGTGTC |
| D15 Mit 29 | 15 | 180 | 160 | -20 | TCACTCTCCACCTCCCAGAT | GTCTGCTTTGTCATAGTGCTGG |
| D16 Mit 139 | 16 | 170 | 150 | -20 | GTATGTAAGGAATGGTCAAATTCTTG | TCATTGTGATTGTGAAAGAATGC |
| D16 Mit 57a | 16 | 100 | 120 | 20 | AAAAAATTTTAAACCATGTGAATGT | TGAAGTTTATTATGAGTTGAATCATGC |
| D17 Mit 123 | 17 | 160 | 140 | -20 | CACAAGGAGGGAGCCTGTAG | CACCGTAAGAGTCTAATAATAAG |
| D17 Mit 19 | 17 | 200 | 180 | -20 | GAGCTGGTAAATGCTTTGGC | TTGAGTACCCCGTACTTGCC |
| D17 Mit 93 | 17 | 140 | 155 | 15 | TGTCCTTCGAGTGTTTGTGTG | TCCCCGGTGAATGAGTTATC |
| D18 Mit 124 | 18 | 130 | 140 | 10 | CCCAAATGGGGTGTCTTTTA | CTGCCACACATTTGTGTGTAT |
| D18 Mit 184 | 18 | 125 | 170 | 45 | CACACATGTGTAGGTAGGTAGGTAGG | CGCACAAGGACTACTGAAACA |
| D18 Mit 55 | 18 | 220 | 225 | 5 | ACAGATGTTCCCCAGCATTC | TGAGTGTGAGATCAGCCTG |
| D18 Mit 94 | 18 | 140 | 155 | 15 | TCACCTAGGACCCCCCTC | AAGTAGTGAGAGGCCACCACA |
| D19 Mit 10 | 19 | 150 | 140 | -10 | GCCTTTAAGCCAGTCAAGAC | CCAGTCTGGACTTGTGAATG |
| D19 Mit 19 | 19 | 190 | 160 | -30 | CCTGTGTCCATACAGGCTCA | ACCATATCAGGAAGCACCATG |

**Table S2: Echocardiographic parameters for C57BL/6JRj, C57*^Lmna^* ^p.H222P^, 129S2/svPasCrl and 129*^Lmna^* ^p.H222P^ mice**

| **Genotype** | **C57BL/6JRj** | **C57*^Lmna^* ^p.H222P^** | **129S2/svPasCrl** | **129*^Lmna^* ^p.H222P^** |
| --- | --- | --- | --- | --- |
| n | 8 | 6 | 6 | 9 |
| age, months | 3 | 3 | 3 | 3 |
| Heart rate, bpm | 577.5 ± 30.48 | 584.9 ± 32.35 | 593.9 ± 28.54 | 572.5 ± 25.89 |
| IVSd, mm | 0.62 ± 0.04 | 0.60 ± 0.00 | 0.70 ± 0.00 | 0.67 ± 0.04 |
| LVPWd, mm | 0.67 ± 0.10 | 0.65 ± 0.10 | 0.68 ± 0.04 | 0.62 ± 0.08 |
| LVDd, mm | 3.36 ± 0.19 | 3.73 ± 0.27 | 3.63 ± 0.19 | 4.02 ± 0.64 |
| IVSs, mm | 1.07 ± 0.08 | 1.11 ± 0.11 | 1.20 ± 0.06 | 1.13 ± 0.10 |
| LVPWs, mm | 1.12 ± 0.12 | 1.10 ± 0.06 | 1.15 ± 0.05 | 1.09 ± 0.12 |
| LVDs, mm | 1.83 ± 0.09 | 2.10 ± 0.20 | 1.98 ± 0.11 | 2.35 ± 0.71 |
| FS, % | 45.17 ± 1.07 | 43.54 ± 1.57 | 45.95 ± 1.19 | 42.62 ± 7.70 |
|  |  |  |  |  |
| **Genotype** | **C57BL/6JRj** | **C57*^Lmna^* ^p.H222P^** | **129S2/svPasCrl** | **129*^Lmna^* ^p.H222P^** |
| n | 8 | 6 | 12 | 9 |
| age, months | 4 | 4 | 4 | 4 |
| Heart rate, bpm | 595.9 ± 35.36 | 614.5 ± 25.74 | 562.8 ± 30.07 | 594.7 ± 32.6 |
| IVSd, mm | 0.63 ± 0.05 | 0.60 ± 0.00 | 0.68 ± 0.04 | 0.64 ± 0.07 |
| LVPWd, mm | 0.63 ± 0.07 | 0.61 ± 0.09 | 0.70 ± 0.07 | 0.68 ± 0.08 |
| LVDd, mm | 3.41 ± 0.29 | 3.73 ± 0.10 | 3.73 ± 0.20 | 4.01 ± 0.60 |
| IVSs, mm | 1.11 ± 0.06 | 1.03 ± 0.12 | 1.18 ± 0.08 | 1.06 ± 0.17 |
| LVPWs, mm | 1.15 ± 0.10 | 1.06 ± 0.08 | 1.11 ± 0.08 | 1.09 ± 0.12 |
| LVDs, mm | 1.86 ± 0.19 | 2.17 ± 0.08 | 2.04 ± 0.14 | 2.62 ± 0.69^*^ |
| FS, % | 45.39 ± 1.41 | 42.21 ± 0.87 ^O^ | 45.6 ± 1.11 | 35.39 ± 7.09^***^ |
|  |  |  |  |  |
| **Genotype** | **C57BL/6JRj** | **C57*^Lmna^* ^p.H222P^** | **129S2/svPasCrl** | **129*^Lmna^* ^p.H222P^** |
| n | 8 | 6 | 12 | 9 |
| age, months | 5 | 5 | 5 | 5 |
| Heart rate, bpm | 618.7 ± 17.85 | 599.2 ± 24.31 | 569.9 ± 24.48 | 596.7 ± 36.26 |
| IVSd, mm | 0.62 ± 0.05 | 0.63 ± 0.05 | 0.71 ± 0.03 | 0.68 ± 0.04 |
| LVPWd, mm | 0.60 ± 0.10 | 0.67 ± 0.10 | 0.66 ± 0.05 | 0.62 ± 0.07 |
| LVDd, mm | 3.50 ± 0.26 | 3.73 ± 0.22 | 4.03 ± 0.31 | 4.18 ± 0.48 |
| IVSs, mm | 1.14 ± 0.05 | 0.98 ± 0.07 | 1.21 ± 0.08 | 0.98 ± 0.12^***^ |
| LVPWs, mm | 1.10 ± 0.09 | 0.95 ± 0.10 | 1.08 ± 0.07 | 0.98 ± 0.16 |
| LVDs, mm | 1.93 ± 0.14 | 2.48 ± 0.19 | 2.28 ± 0.27 | 3.25 ± 0.91^*^ |
| FS, % | 44.88 ± 1.56 | 33.3 ± 2.28^#^ | 43.34 ± 2.52 | 29.12 ± 7.21^**^ |
|  |  |  |  |  |

| **Genotype** | **C57BL/6JRj** | **C57*^Lmna^* ^p.H222P^** | **129S2/svPasCrl** | **129*^Lmna^* ^p.H222P^** |
| --- | --- | --- | --- | --- |
| n | 8 | 6 | 12 | 8 |
| age, months | 6 | 6 | 6 | 6 |
| Heart rate, bpm | 602.2 ± 15.76 | 601.9 ± 28.25 | 578.9 ± 31.12 | 555.3 ± 55.56 |
| IVSd, mm | 0.70 ± 0.00 | 0.60 ± 0.00 | 0.73 ± 0.05 | 0.62 ± 0.07^**^ |
| LVPWd, mm | 0.81 ± 0.08 | 0.65 ± 0.06 | 1.12 ± 1.53 | 0.56 ± 0.16 |
| LVDd, mm | 3.67 ± 0.22 | 4.02 ± 0.32 | 4.17 ± 0.23 | 4.89 ± 0.64 |
| IVSs, mm | 1.17 ± 0.07 | 0.93 ± 0.05 | 1.25 ± 0.07 | 0.90 ± 0.11^***^ |
| LVPWs, mm | 1.31 ± 0.11 | 0.97 ± 0.05 | 1.15 ± 0.09 | 0.67 ± 0.17^**^ |
| LVDs, mm | 2.04 ± 0.16 | 2.767 ± 0.29^#^ | 2.41 ± 0.28 | 4.06 ± 0.89^**^ |
| FS, % | 44.29 ± 1.75 | 30.61 ± 3.28^#^ | 42.16 ± 3.59 | 17.97 ± 7.96^***^ |
|  |  |  |  |  |
| **Genotype** | **C57BL/6JRj** | **C57*^Lmna^* ^p.H222P^** | **129S2/svPasCrl** | **129*^Lmna^* ^p.H222P^** |
| n | 8 | 6 | 12 | 4 |
| age, months | 7 | 7 | 7 | 7 |
| Heart rate, bpm | 600.5 ± 17 | 607.6 ± 17.34 | 566.8 ± 30.30 | 557.7 ± 52.92 |
| IVSd, mm | 0.66 ± 0.05 | 0.60 ± 0.00 | 0.78 ± 0.06 | 0.65 ± 0.06 |
| LVPWd, mm | 0.72 ± 0.09 | 0.62 ± 0.12 | 0.71 ± 0.07 | 0.55 ± 0.06 |
| LVDd, mm | 3.67 ± 0.17 | 4.03 ± 0.33 | 3.98 ± 0.24 | 4.87 ± 0.57 |
| IVSs, mm | 1.15 ± 0.07 | 0.92 ± 0.07 | 1.27 ± 0.10 | 0.95 ± 0.06^**^ |
| LVPWs, mm | 1.3 ± 0.09 | 0.90 ± 0.15 | 1.20 ± 0.10 | 0.75 ± 0.13^**^ |
| LVDs, mm | 2.05 ± 0.17 | 2.92 ± 0.36^##^ | 2.20 ± 0.17 | 3.92 ± 0.64^**^ |
| FS, % | 44.13 ± 1.52 | 27.86 ± 3.26^#^ | 44.5 ± 1.96 | 19.78 ± 3.94^***^ |
|  |  |  |  |  |
| **Genotype** | **C57BL/6JRj** | **C57*^Lmna^* ^p.H222P^** | **129S2/svPasCrl** | **129*^Lmna^* ^p.H222P^** |
| n | 8 | 6 | 12 | 2 |
| age, months | 8 | 8 | 8 | 8 |
| Heart rate, bpm | 622.1 ± 11.17 | 593.8 ± 29.67 | 599.6 ± 17.50 | 502.8 ± 90.75 |
| IVSd, mm | 0.66 ± 0.05 | 0.60 ± 0.06 | 0.69 ± 0.03 | 0.60 ± 0.00^*^ |
| LVPWd, mm | 0.71 ± 0.06 | 0.65 ± 0.05 | 0.69 ± 0.05 | 0.65 ± 0.07 |
| LVDd, mm | 3.60 ± 0.19 | 4.43 ± 0.76^#^ | 4.17 ± 0.39 | 5.10 ± 0.00^*^ |
| IVSs, mm | 1.11 ± 0.06 | 0.95 ± 0.15 | 1.23 ± 0.12 | 0.85 ± 0.07^*^ |
| LVPWs, mm | 1.16 ± 0.07 | 0.91 ± 0.14^#^ | 1.11 ± 0.03 | 0.75 ± 0.07^*^ |
| LVDs, mm | 1.97 ± 0.13 | 3.27 ± 0.95^#^ | 2.54 ± 0.61 | 4.35 ± 0.21^*^ |
| FS, % | 44.96 ± 1.53 | 24.12 ± 7.69^#^ | 39.68 ± 8.36 | 15.1 ± 2.92^*^ |
|  |  |  |  |  |

| **Genotype** | **C57BL/6JRj** | **C57*^Lmna^* ^p.H222P^** |  |  |
| --- | --- | --- | --- | --- |
| n | 4 | 3 |  |  |
| age, months | 10 | 10 |  |  |
| Heart rate, bpm | 609.5 ± 36.36 | 586.1 ± 14.07 |  |  |
| IVSd, mm | 0.67 ± 0.05 | 0.57 ± 0.05^#^ |  |  |
| LVPWd, mm | 0.70 ± 0.00 | 0.60 ± 0.09^###^ |  |  |
| LVDd, mm | 3.77 ± 0.27 | 5.07 ± 0.14^#^ |  |  |
| IVSs, mm | 1.20 ± 0.13 | 0.73 ± 0.05^###^ |  |  |
| LVPWs, mm | 1.20 ± 0.18 | 0.80 ± 0.09^##^ |  |  |
| LVDs, mm | 2.12 ± 0.16 | 4.37 ± 0.19^###^ |  |  |
| FS, % | 43.07 ± 4.59 | 14.27 ± 1.97^###^ |  |  |

n: number of mice; IVS, inter ventricular septum; LVPW, left ventricular posterior wall; LVD, left ventricular diameter; FS, fractional shortening; s, systole; d, diastole. Values are presented as mean ± SD. Multiple group comparison was performed with Kruskal Wallis test with Dunn’s test post-test

**p*≤0.05, ***p*≤0.005 and ****p*≤0.0005 between 129*^Lmna^* ^p.H222P^ mice and 129S2/svPasCrl mice.

^#^*p*≤0.05, ^##^*p*≤0.005 and ^###^*p*≤0.0005 between C57*^Lmna^* ^p.H222P^ and C57BL/6JRj mice.

^o^*p*≤0.05 between C57*^Lmna^* ^p.H222P^ and 129*^Lmna^* ^p.H222P^ mice.

**Table S3: Electrocardiographic parameters for C57BL/6JRj, C57*^Lmna^* ^p.H222P^, 129S2/svPasCrl and 129*^Lmna^* ^p.H222P^**

| **Genotype** | **C57BL/6JRj** | **C57*^Lmna^* ^p.H222P^** | **129S2/svPasCrl** | **129*^Lmna^* ^p.H222P^** |
| --- | --- | --- | --- | --- |
| n | 8 | 6 | 6 | 9 |
| age, months | 3 | 3 | 3 | 3 |
| RR, ms | 80.74 ± 2.24 | 82.83 ± 1.65 | 86.48 ± 3.26 | 91.53 ± 6.24^*^ |
| PR, ms | 30.97 ± 1.51 | 31.68 ± 0.77 | 30.14 ± 1.79 | 30.79 ± 2.22 |
| QRS, ms | 12.01 ± 0.31 | 12.21 ± 0.37 | 12.88 ± 098 | 13.65 ± 1.57 |
|  |  |  |  |  |
| **Genotype** | **C57BL/6JRj** | **C57*^Lmna^* ^p.H222P^** | **129S2/svPasCrl** | **129*^Lmna^* ^p.H222P^** |
| n | 8 | 6 | 11 | 7 |
| age, months | 4 | 4 | 4 | 4 |
| RR, ms | 79.22 ± 2.18 | 83.12 ± 7.00 | 96.62±10.42 | 98.27±13.58 |
| PR, ms | 29.6 ± 1.47 | 31.7 ± 3.09 | 30.84 ± 2.40 | 29.43 ± 1.56 |
| QRS, ms | 12.15 ± 0.41 | 12.43 ± 0.45 | 12.87± 0.62 | 14.51 ± 2.30 |
|  |  |  |  |  |
| **Genotype** | **C57BL/6JRj** | **C57*^Lmna^* ^p.H222P^** | **129S2/svPasCrl** | **129*^Lmna^* ^p.H222P^** |
| n | 7 | 6 | 10 | 9 |
| age, months | 5 | 5 | 5 | 5 |
| RR, ms | 81.32 ± 3.34 | 82.04 ± 1.25 | 87.89 ± 6.11 | 94.16 ± 11.11^*^ |
| PR, ms | 31.10 ± 2.29 | 30.77 ± 1.10 | 30.22 ± 1.92 | 33.16 ± 3.85 |
| QRS, ms | 11.96 ± 0.24 | 12.38 ± 0.17 | 12.6 ± 0.70 | 14.48 ± 2.60 |
|  |  |  |  |  |
| **Genotype** | **C57BL/6JRj** | **C57*^Lmna^* ^p.H222P^** | **129S2/svPasCrl** | **129*^Lmna^* ^p.H222P^** |
| n | 7 | 6 | 12 | 8 |
| age, months | 6 | 6 | 6 | 6 |
| RR, ms | 78.84 ± 2.20 | 82.32 ± 1.59^o^ | 93.5 ± 9.24 | 112.3 ± 13.35^**^ |
| PR, ms | 30.86 ± 1.32 | 31.61 ± 1.63 | 30.64 ± 1.63 | 33.81 ± 3.65 |
| QRS, ms | 11.8 ± 0.26 | 12.65 ± 0.16 | 12.91 ± 0.49 | 17.57 ± 4.71 |
|  |  |  |  |  |
| **Genotype** | **C57BL/6JRj** | **C57*^Lmna^* ^p.H222P^** |  |  |
| n | 8 | 6 |  |  |
| age, months | 7 | 7 |  |  |
| RR, ms | 78.54 ± 1.70 | 87.99 ± 5.38^##^ |  |  |
| PR, ms | 31.13 ± 2.14 | 34.65 ± 3.44^#^ |  |  |
| QRS, ms | 12.07 ± 0.48 | 13.97 ± 2.59^##^ |  |  |

n: number of mice; RR, duration of the RR intervals; PR duration of PR intervals; QRS, duration of the QRS complexes. Values are presented as mean ± SD. Multiple group comparison was performed with Kruskal Wallis test with Dunn’s test post-test.

**p*≤0.05 and ***p*≤0.005 between 129*^Lmna^* ^p.H222P^ mice and 129S2/svPasCrl mice.

^#^*p*≤0.05, ^##^*p*≤0.005 between C57*^Lmna^* ^p.H222P^ and C57BL/6JRj mice.

**Table S4: Electrocardiographic parameters analyzed by Kruskal-Wallis multiple comparison test *p* values**

| ECG parameter | age, months | *p* value | | |
| --- | --- | --- | --- | --- |
|  |  | C57BL/6JRj vs C57*^Lmna^* ^p.H222P^ | 129S2/svPasCrl vs 129*^Lmna^* ^p.H222P^ | C57*^Lmna^* ^p.H222P^ vs 129*^Lmna^* ^p.H222P^ |
| RR | 3 | >0.9999 | >0.9999 | 0.0359 |
|  | 4 | >0.9999 | >0.9999 | 0.0925 |
|  | 5 | >0.9999 | >0.9999 | 0.015 |
|  | 6 | >0.9999 | 0.3694 | 0.0041 |
|  | 7 | 0.0013 | ND | ND |
| PR | 3 | >0.9999 | >0.9999 | >0.9999 |
|  | 4 | 0.4299 | 0.9506 | >0.9999 |
|  | 5 | >0.9999 | 0.4278 | >0.9999 |
|  | 6 | >0.9999 | 0.1748 | >0.9999 |
|  | 7 | 0.0426 | ND | ND |
| QRS | 3 | >0.9999 | >0.9999 | 0.0549 |
|  | 4 | >0.9999 | >0.9999 | 0.1173 |
|  | 5 | 0.4822 | 0.7545 | 0.9003 |
|  | 6 | 0.1758 | 0.8549 | 0.3165 |
|  | 7 | 0.005 | ND | ND |
